# Use of patient diaries in conjunction with standard reporting methods: duplication of data or a valuable resource?

**DOI:** 10.1186/1745-6215-16-S2-P183

**Published:** 2015-11-16

**Authors:** Janet Dunn, Bill Harris, Gulnaz Iqbal, Jill Wood, Pankaj Mistry, Joanne O'Beirne-Elliman, Stella Bowcock, Mark Drayson

**Affiliations:** 1 University of Warwick, Coventry, West Midlands, UK; 2 King’s College Hospital NHS Foundation Trust, London, UK; 3 University of Birmingham, Birmingham, West Midlands, UK

## 

There is a high early death rate in patients with myeloma with the single biggest cause being infection. Antibiotic prophylaxis is likely to be the most effective measure to prevent early death in this patient population. TEAMM is a randomised, double-blind, placebo-controlled multi-centre phase III clinical trial seeking to tackle early morbidity and mortality in patients with myeloma by assessing the benefit of antibiotic prophylaxis and its effect on healthcare associated infections. The main endpoint is the number of febrile episodes suffered in the first 12 weeks from randomisation (defined as an oral temperature of ≥38°C treated with anti-infectives). Secondary outcomes include the number of clinically documented total infections, episodes of severe sepsis and suspected infections. Occurrence of febrile episodes is captured at clinic visits every 4 weeks. Furthermore, patients were supplied with diary cards and thermometers.

TEAMM aims to recruit 800 patients randomising to either levofloxacin or placebo. The first patient was recruited in August 2012, with 723 patients randomised to-date, of these 402 have completed treatment and 1139 diaries have been returned. Review by an independent clinician cross-referencing patient diaries with CRFs demonstrated the data collected were strengthened through the use of patient diaries. Patient diaries can enhance data quality, and when used alongside usual reporting methods can optimise data accuracy and robustness of study findings.

## Funding

Project funded by NIHR HTA programme (08/116/69). Views expressed are those of the authors and not those of the HTA programme, NIHR, NHS or the Department of Health.

